# Nighttime Image Stitching Method Based on Image Decomposition Enhancement

**DOI:** 10.3390/e25091282

**Published:** 2023-08-31

**Authors:** Mengying Yan, Danyang Qin, Gengxin Zhang, Huapeng Tang, Lin Ma

**Affiliations:** 1 Department of Electronic Engineering, Heilongjiang University, Harbin 150080, China; 2211723@s.hlju.edu.cn (M.Y.); 2211779@s.hlju.edu.cn (G.Z.); 2211781@s.hlju.edu.cn (H.T.); 2 National Mobile Communications Research Laboratory, Southeast University, Nanjing 210096, China; 3 Department of Electronics and Information Engineering, Harbin Institute of Technology, Harbin 150080, China; malin@hit.edu.cn

**Keywords:** night image stitching, image enhancement, feature extraction, edge enhancement

## Abstract

Image stitching technology realizes alignment and fusion of a series of images with common pixel areas taken from different viewpoints of the same scene to produce a wide field of view panoramic image with natural structure. The night environment is one of the important scenes of human life, and the night image stitching technology has more urgent practical significance in the fields of security monitoring and intelligent driving at night. Due to the influence of artificial light sources at night, the brightness of the image is unevenly distributed and there are a large number of dark light areas, but often these dark light areas have rich structural information. The structural features hidden in the darkness are difficult to extract, resulting in ghosting and misalignment when stitching, which makes it difficult to meet the practical application requirements. Therefore, a nighttime image stitching method based on image decomposition enhancement is proposed to address the problem of insufficient line feature extraction in the stitching process of nighttime images. The proposed algorithm performs luminance enhancement on the structural layer, smoothes the nighttime image noise using a denoising algorithm on the texture layer, and finally complements the texture of the fused image by an edge enhancement algorithm. The experimental results show that the proposed algorithm improves the image quality in terms of information entropy, contrast, and noise suppression compared with other algorithms. Moreover, the proposed algorithm extracts the most line features from the processed nighttime images, which is more helpful for the stitching of nighttime images.

## 1. Introduction

Images can help humans understand the world most intuitively. It is an important channel for human and nature to conduct information interaction with nature and the basis of the research of computer vision. With the development of computer vision technology, a limited perspective can no longer meet people’s needs, and a broader perspective and richer information demand under the field of views are becoming increasingly urgent. When people obtain high-resolution and wide-viewed images, because wide-angle lenses still have perspective limitations, fish-eyed lenses are serious, panoramic cameras are expensive and complicated, and there are great limitations for obtaining broad-vision images in hardware. Therefore, digital image stitching technology shows unique advantages. Image stitching is the technology of two or more images taken in a certain environment through image processing into a wider panoramic image technology. This technology can also take into account the high field of view and high distortion, thereby meeting people’s needs for scene observation, and is used in the fields of autonomous driving, virtual tourism, and video surveillance [1].

The main steps of the image stitching technique include image pre-processing, feature extraction, feature matching, and image fusion. Previous splicing algorithms focused on improving the extraction and alignment of feature points, however, in recent years, more and more researchers have begun to introduce linear features to assist in the alignment. Real-life scenes are full of rich linear structures, and it is obviously very positive to protect the linear structures in image stitching. Especially in some low-texture images where it is difficult to extract enough effective matching points, the line features can compensate well to obtain more matching information. Moreover, the human eye has a good perception of linear structures, and matching linear structures in image stitching, building alignment relationships between images, and thus protecting linear structures is also of great benefit for enhancing the natural look and feel of the stitching results.

**Figure 1:**
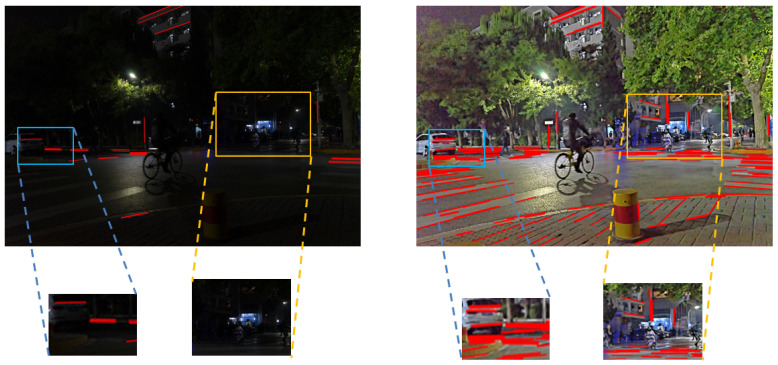
Line feature extraction effect before and after night image enhancement.

Night is one of the important scenes of human life, and the night image stitching technology can be applied to security monitoring, night intelligent driving, and other fields. However, most of the current mature image stitching techniques use images with good lighting conditions, while image stitching techniques in low-light scenes such as at night are not yet perfect. The night scene light source is complex, containing both weak natural light (such as moonlight, starlight, etc.) and light generated by artificial light sources (such as streetlights, headlights, etc.). Within the scene of artificial light source, the influence produced by natural illumination becomes weaker. The average brightness of the night images containing artificial light sources is improved compared with the night images under natural illumination fields. However, since artificial light sources can only affect local scenes, it causes the problems of uneven luminance distribution, low visibility of darker luminance areas, and serious loss of details in the captured night images. Moreover, the brightness of the area near the light source is high, and the detail information is also masked, making the degradation of the night image serious. When feature extraction is performed on nighttime images, it is found that the line features of the images are mainly concentrated in the bright areas of the images. When the image is matched, the stitching fails due to the lack of feature information of the image when the bright area is in the non-stitching overlap area. In order to improve the image quality and stitching success rate, we use the enhancement technique to pre-process the nighttime images. The effect of line feature extraction before and after the nighttime image enhancement is shown in Figure 1.

## 2. Related Work

In this paper, we use enhancement algorithms to pre-process the images to be stitched at night for the purpose of complementing the image line features while improving the night image quality. The low-light image enhancement algorithm mainly enhances the overall contrast and brightness of the image by improving the brightness of dark areas and suppressing the gray value of overly bright areas. Low-illumination image enhancement, a classical problem in the field of digital image processing, has been evolving for a long time. Common enhancement methods for low-illumination color images include Retinex theory-based methods, histogram equalization-based methods, and image fusion-based enhancement methods.

Multi-scale Retinex (MSR) [2] and multi-scale Retinex with color restoration (MSRCR) [3] are representative Retinex algorithms. However, these algorithms are prone to color distortion, halation, and over-enhancement. Adaptive multi-scale Retinex (AMSR) [4] is a weighting strategy based on the SSR (single scale retinex) algorithm. Tang et al. [5] used the Retinex model in the Y channel to enhance the luminance, but the image edges were blurred due to denoising and smoothing, resulting in a decrease in the contrast of the image. Based on Retinex theory, Guo et al. [6] proposed a detail enhancement method based on guided filtering to optimize the reflectance map and generate enhanced ocean images. Wang et al. [7] proposed a color image correction method based on nonlinear function transform to improve the image brightness, but the method parameters need to be set manually and the image processing effect depends on the selection of parameters.

Histogram equalization can effectively improve the brightness and contrast of the image, and the principle is simple and easy to implement. The dual histogram [8] equalization method is an algorithm to adjust the brightness of the input image. Lu et al. [9] proposed an image enhancement method based on adaptive detail equalization, which enhances the image brightness and contrast by adaptive dual-interval histogram equalization. Veluchamy and Subramani [10] used a new adaptive gamma correction method to enhance the image contrast while employing a weighted histogram distribution to maintain the natural color and detail of the image. Liu et al. [11] proposed an adaptive contrast enhancement method based on a histogram correction framework to improve the local detail features of the image by spatially segmenting the original image. Gautam and Tiwari [12] combined the limiting dual histogram equalization with adaptive gamma correction to effectively enhance low-contrast color images.

Fusion-based image enhancement techniques can recover image details very well. Li et al. [13] used the wavelet image fusion technique to highlight the details of the image so that the enhanced image has higher clarity and visual effect. Ren et al. [14] proposed an effective low-light image enhancement method to maintain naturalness and obtain a natural image of the picture. Fu et al. [15] proposed a fusion-based low-light image enhancement method to improve image brightness and contrast by selecting appropriate inputs and weights from the estimated illumination. Lee et al. [16] adaptively segmented the input image into a dark background region and bright background region. Subsequently, contrast stretching is performed only in the dark region, which can effectively enhance the contrast of backlit images without oversaturation problems but requires precise segmentation of the dark regions of the image. Lu and Zhang [17] proposed a highly adaptive two-branch fusion strategy to enhance weakly illuminated images with targeted enhancement for slightly distorted and severely distorted images and further proposed an adaptive fusion mechanism.

In recent years, intelligent algorithms have developed rapidly and have also been applied to image enhancement. Qian et al. [18] proposed an adaptive image enhancement algorithm based on visual saliency. This algorithm introduced a cuckoo search algorithm and a bilateral gamma adjustment function in the HIS color space to improve the overall brightness of the image. Kanmani and Narasimhan [19] established a population intelligence-based color image contrast enhancement algorithm that uses an adaptive gamma correction factor selected by a particle swarm algorithm (PSO) to improve the image entropy and enhance the image details. Li et al. [20] proposed an adaptive chaotic particle swarm optimization algorithm (ACPSO) combined with gamma correction to iteratively find the best image for global brightness adjustment. The problem of low brightness and low contrast of low-illumination color images is effectively improved.

**Figure 2:**
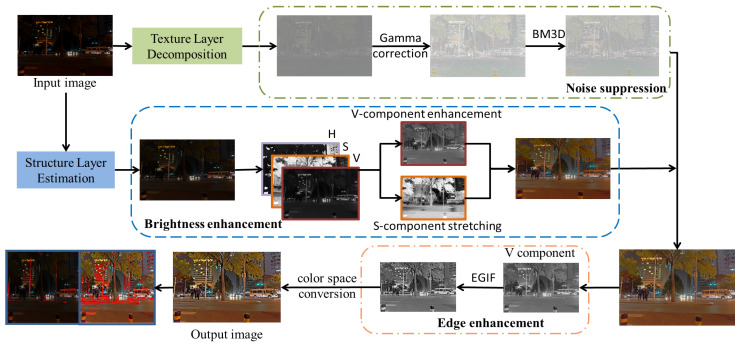
Overall framework of proposed enhancement method.

These algorithms are commonly validated using images from publicly available datasets and are not validated on actual collected low-light images. Since the light in the nighttime environment tends to be very scattered, it results in multiple dark and bright regions in different locations of the image. In contrast, the dark and bright regions of low-illumination images tend to be more concentrated, and ordinary low-illumination image enhancement algorithms cannot handle nighttime images well. In addition, the dark areas of nighttime images usually contain a lot of noise. When enhancing a nighttime image, the amplified noise while restoring the image details seriously affects the image quality. Moreover, these dark areas often have rich linear structures, and when stitching the nighttime images, the point features and line features of the images are hidden and difficult to extract, which affects the subsequent stitching. Therefore, we propose a nighttime image enhancement method based on image decomposition in which the image is decomposed by the rolling guidance filter to obtain the structural layer and texture layer, followed by a brightness enhancement function to improve the brightness of the V component of the structural layer and saturation stretching of the S component to obtain the image of the structural layer after brightness enhancement. After that, the texture layer containing noise is smoothed and denoised, and the enhanced structural layer and the smoothed texture layer are fused. To highlight the edge information of the image, the contrast of the fused image is enhanced to highlight the edge detail information of the image. The proposed algorithm obtains higher quality nighttime images with richer details and sharper edges. When line features are extracted from the enhanced images, line features are extracted from the dark light areas of the images, which proves that the present algorithm also achieves greater improvement in line feature extraction and improves the stitching accuracy. The framework of the proposed algorithm is shown in Figure 2.

The remaining contents of this paper are arranged as follows. Section 3 presents the proposed enhancement algorithm. Section 4 contains the experimental results and discussion. Finally, Section 5 gives the conclusion.

## 3. The Proposed Night Image Enhancement Method

### 3.1. Image Decomposition Model

Most low-illumination image enhancement algorithms inevitably enlarge the image noise while enhancing the brightness, which will affect the quality of the image. The image decomposition framework can effectively solve this problem. We use the image decomposition method to decompose the night image into the structure layer and texture layer. The structural layer component is the smooth renderings of the input image. We increase the brightness of the structure layer to avoid noise amplification due to enhancement of brightness.

Input image *I* consists of a large-scale structure layer $I_{S}$ and fine-scale texture layer $I_{T}$. The expression is shown below.

(1) $$\begin{array}{c} I=I_{S}+I_{T} \end{array}$$

**Figure 3:**
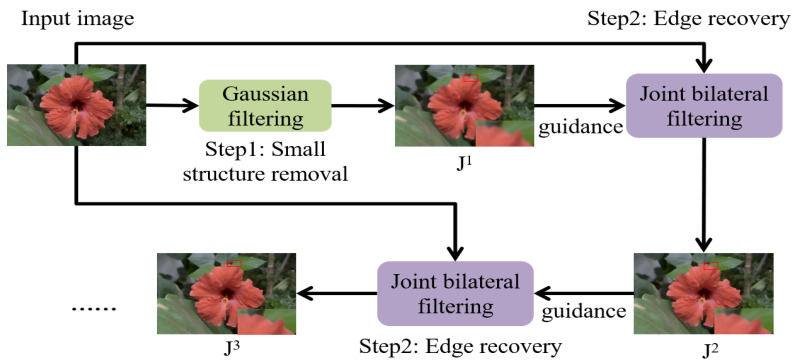
Principle diagram of the rolling guidance filter.

In order to obtain the structure layer, we choose the rolling guidance filter to smooth the night image. The rolling guidance filter is a new edge-retaining filter proposed by Zhang [21]. The difference from other edges to retain the filter is that the rolling guidance filter can be iterated into the implementation process, which can achieve rapid convergence. The implementation of this filter is simple and fast, and the scale separation is achieved in the space-overlapping characteristics. The rolling guidance filter includes two processes: small structure removal and edge recovery. The schematic diagram is shown in Figure 3.

Small structure removal is achieved through Gaussian filtering. A small structure is usually detailed information, including information such as texture, noise, and small goals. The filter expression is as follows.

(2) $$\begin{array}{cc} G(p) & =\frac{1}{\;K_{p}}\sum_{q\in N(p)}\exp\left(-\frac{{∥p-q∥}^{2}}{2\sigma_{s}^{2}}\right)I\left(q\right) \end{array}$$

where

(3) $$\begin{array}{cc} K(p) & =\sum_{q\in N(p)}\exp\left(-\frac{{∥p-q∥}^{2}}{2\sigma_{s}^{2}}\right) \end{array}$$

where *I* represents the input image, *G* represents the output image, *p* and *q* are pixels in the input image *I*, and N(p) is a neighboring pixel set centered on pixel *p*. ‖p−q‖ indicates the distance between the two pixels. $\sigma_{s}$ is the standard deviation in the neighborhood, which is a scale threshold. When the structure in the image is greater than $\sigma_{s}$, it will be retained, otherwise it will be eliminated.

The edge recovery uses iterative form to achieve continuously updating the output image *J*. We denote $J^{t+1}$ as the result of the *t*-th iteration. The initial value $J^{1}$ is obtained by the Gaussian filtering shown in Equation (2). The output image $J^{t+1}$ of the *t*-th iteration is obtained by the input image *I* and the previous iteration output image $J^{t}$ through combining bilateral filtering methods, as shown in the following formula.

(4) $$\begin{array}{cc} J^{t+1}\left(p\right) & =\frac{1}{\;K_{p}}\sum_{q\in N(p)}\exp\left(-\frac{{∥p-q∥}^{2}}{2\sigma_{s}^{2}}-\frac{{∥J^{t}\left(p\right)-J^{t}\left(q\right)∥}^{2}}{2\sigma_{r}^{2}}\right)I\left(q\right) \end{array}$$

where

(5) $$\begin{array}{cc} K_{p} & =\sum_{q\in N(p)}\exp\left(-\frac{{∥p-q∥}^{2}}{2\sigma_{s}^{2}}-\frac{{∥J^{t}\left(p\right)-J^{t}\left(q\right)∥}^{2}}{2\sigma_{r}^{2}}\right) \end{array}$$

Equation (4) can be understood as a guidance image with the structure of $J^{t}$ and can smoothly enter the filter form of image *I*. The process of constantly updating the guidance image is called rolling guidance filtering.

The nighttime image is processed by a rolling guide filter to obtain a structural layer image, as shown in Equation (6). RGF represents rolling guidance filter, *t* is the number of iterations. The texture layer is obtained by the difference between the original image and the structure layer, as shown in Equation (7).

(6) $$\begin{array}{c} I_{S}=RGF\left(I,\sigma_{s},\sigma_{r},t\right) \end{array}$$

(7) $$\begin{array}{c} I_{T}=I-I_{S} \end{array}$$

**Figure 4:**
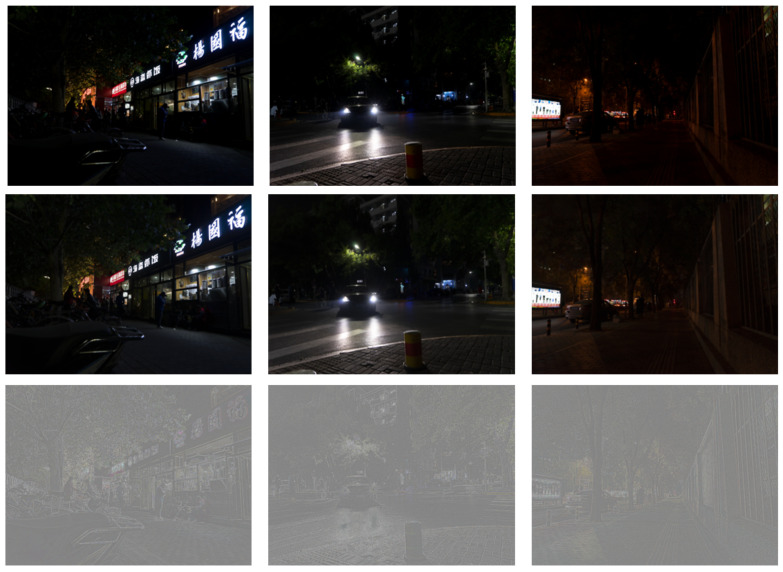
Top row: original image; middle row: structure layer; bottom row: texture layer.

The effect of image decomposition is shown in Figure 4. In order to make the texture layer easy to observe, a gamma transform is used to improve the brightness of the texture layer. The texture layer is offset by 0.5 and then displayed.

### 3.2. Brightness Enhancement of Structural Layer

The enhancement processing of RGB color space easily causes the color distortion of the image, so we choose the HSV color space closer to the human visual expectations to enhance the image. We convert the RGB space of the image to HSV space and obtain three components, namely H (hue), S (saturation), and V (luminance), respectively, as $I_{h}\left(x,y\right)$, $I_{s}\left(x,y\right)$, and $I_{v}\left(x,y\right)$.

In the Retinex-based image enhancement algorithm, Gaussian filtering and bilateral filtering are usually used as a surround function to estimate the light component [22]. Gaussian filtering can be used to extract light components, but as the filter window increases, the calculation complexity will increase significantly. When the bilateral filtering is treated with color images, the gradient reversal is generated near the edge of the object in the image, causing the halo to appear [23]. This article uses a linear guidance filter with smooth and bordering functions to estimate the light component and uses brightness component $I_{v}\left(x,y\right)$ as input images and guide images. Considering that the slow change of light in most areas leads to sudden changes in local area luminance, the luminance components are processed by two guidance filters and fused together by averaging and weighting as the final light component estimate. The expression is shown below.

(8) $$\begin{array}{cc} F_{1}\left(x,y\right) & =GF_{r1,\lambda 1}\left[I_{v}\left(x,y\right),I_{v}\left(x,y\right)\right] \end{array}$$

(9) $$\begin{array}{cc} F_{2}\left(x,y\right) & =GF_{r2,\lambda 2}\left[I_{v}\left(x,y\right),F_{1}\left(x,y\right)\right] \end{array}$$

(10) $$\begin{array}{cc} I_{v_{-}gif} & =0.5*F_{1}\left(x,y\right)+0.5*F_{2}\left(x,y\right) \end{array}$$

where $GF_{\left(r,\lambda \right)}$ represents the guided filter function with the window radius as *r* and the regularization parameter as λ. $I_{v_{-}gif}$ denotes the filtered illumination component.

Weber–Fechner’s law shows that the human vision system is a non-linear processing process. The enhanced function is formulated according to Weber–Fechner’s law, and the image obtained is more in line with human vision [7]. Due to the height complexity of the number of calculations, the following functions are used to fit the lighting component.

(11) $$\begin{array}{cc} I_{v}^{\prime } & =\frac{I_{v}\left(255+{\bar{I}}_{s}\times {\bar{I}}_{v}\right)}{\max\left(I_{v},I_{v_{-}gif}\right)+\left({\bar{I}}_{s}\times {\bar{I}}_{v}\right)} \end{array}$$

where ${\bar{I}}_{s}=\frac{1}{N}\sum_{i=1}^{N}I_{s}$, ${\bar{I}}_{v}=\frac{1}{N}\sum_{i=1}^{N}I_{v}$, and *N* is the number of pixels of image $I_{v}$.

After the image brightness is improved, the saturation of the image will be reduced to a certain extent. In order to prevent the impact of brightness on saturation, we quote the adaptive non-linear stretching function proposed by the literature [24] to stretch the saturation of the image. After using this function to process S components, the saturation of the image is higher and the color information of the image is richer.

(12) $$\begin{array}{cc} I_{s}^{\prime } & =\left(0.5+0.5*\frac{\max(R,G,B)+\min(R,G,B)+1}{2*\mathrm{mean}(R,G,B)+1}\right)*I_{s} \end{array}$$

where $I_{s}$ and $I_{s}^{\prime }$ are the saturation of the image before and after stretching. max(R,G,B) indicates maximum value of pixels in R, G, and B color channels. min(R,G,B) refers to minimum value of pixels in the three channels. mean(R,G,B) refers to the average value of pixels in the three color channels.

**Figure 5:**
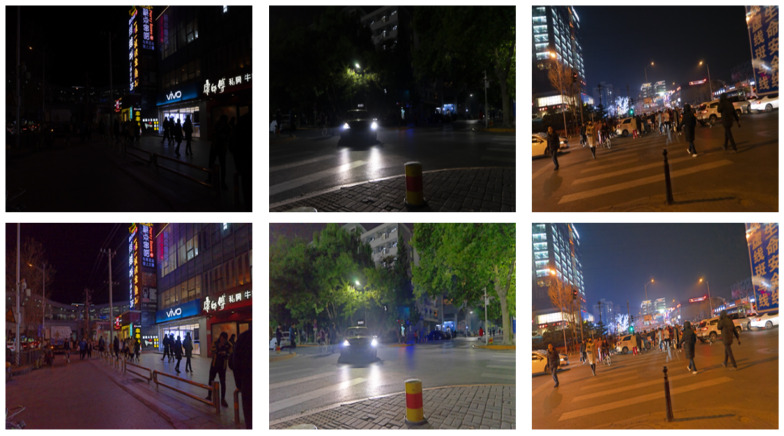
First row: structural layer of the original image; Second row: structural layer after brightness enhancement.

The brightness improvement of the V channel, the saturation stretch of the S component, and the H component remain unchanged. Finally, the enhanced structural layer $I_{S\_E}\left(x,y\right)$ is obtained by converting it to RGB color space. The effect is shown in Figure 5. The enhanced structural layer has greatly improved in terms of brightness and color saturation.

### 3.3. Noise Suppression of Texture Layer

The night image minus the structure layer is used to obtain the original texture layer. The original texture layer is a positive and negative piece of data that is distributed around the zero value, which is rich in detail information and a lot of noise. After obtaining the texture layer, the gamma correction function is used to increase the brightness of the texture layer. Then, the BM3D denoising algorithm [25] is used to smooth the texture layer. The expression is as follows.

(13) $$\begin{array}{c} I_{T\_F}=BM3D\left({\left(I_{T}\right)}^{\frac{1}{g}}\right) \end{array}$$

where $I_{T}$ is the texture of the night image. *g* is a gamma correction parameter, which is generally taken as 2.2. $I_{T\_F}$ is the smooth image layer of the image. After using the BM3D algorithm to deal with the texture layer, the large amount of noise in the image is removed.

### 3.4. Edge Enhancement

The enhanced structural layer $I_{S\_E}$ plus the smooth texture layer $I_{T\_F}$ results in a fusion image R(x,y).

(14) $$\begin{array}{c} R\left(x,y\right)=I_{S\_E}\left(x,y\right)+I_{T\_F}\left(x,y\right) \end{array}$$

In addition, the noise of night images is often distributed in the dark area, but the BM3D performs the same operation on all areas of the image, resulting in excessive smoothness in some areas, and the edge of the image is unclear, which affects line feature extraction. Therefore, it is necessary to enhance the edge of enhanced images. In order to avoid the loss of details of the foreground objects from smoothing, the edge enhancement of the fusion image is enhanced to supplement the detail texture information of the image to make the edge of the image clearer. This article uses effective guide filtering (EGIF) [26] with strong edge maintenance capabilities to smooth the fusion image and then estimate the texture layer of the image. After that, the texture layer is enhanced by the amplifier factor to highlight the edge of the image. The principle of EGIF processing is as follows.

The output image *q* is represented as a linear model related to the guidance image *I*. The formula is as follows:

(15) $$\begin{array}{c} q_{i}=a_{k}I_{i}+b_{k},\;\forall i\in \omega_{k} \end{array}$$

where $q_{i}$ is the gray value of the linear transformation at the *i* pixel in the window $\omega_{k}$ of the image *I*, *k* is the central pixel of the window $\omega_{k}$, and $a_{k}$ and $b_{k}$ are the linear coefficients of the guided image within a local window $\omega_{k}$ with a radius of *r* centered on pixel *k*. The value function settings are as follows:

(16) $$\begin{array}{c} E\left(a_{k},b_{k}\right)=\sum_{i\in \omega_{k}}\left({\left(a_{k}I_{i}+b_{k}-p_{i}\right)}^{2}+\lambda \Gamma a_{k}^{2}\right) \end{array}$$

where

(17) $$\begin{array}{c} \Gamma ={\bar{\sigma }}^{2}=\frac{1}{N}\sum_{k=1}^{N}\sigma_{k}^{2} \end{array}$$

where λ is a parameter used to prevent the regularization parameter of $a_{k}$ becoming too large, which is used to regulate the filter effect of the filter. *p* is the input image, and *N* is the number of pixels in the guidance image *I*. Local linear coefficients $a_{k}$ and $b_{k}$ can be solved through the minimum dwellings:

(18) $$\begin{array}{c} a_{k}=\frac{\frac{1}{N_{\omega_{k}}}\sum_{i\in \omega_{k}}I_{i}p_{i}-\mu_{k}{\bar{p}}_{k}}{\sigma_{k}^{2}+\lambda \Gamma } \end{array}$$

(19) $$\begin{array}{c} b_{k}={\bar{p}}_{k}-a_{k}\mu_{k} \end{array}$$

When GIF is used to enhance image contrast, *I* and *q* are equal, so the following equations are established,

(20) $$\begin{array}{c} \frac{1}{N_{\omega_{k}}}\sum_{i\in \omega_{k}}I_{i}p_{i}-\mu_{k}{\bar{p}}_{k}=\sigma_{k}^{2} \end{array}$$

(21) $$\begin{array}{c} {\bar{p}}_{k}=\mu_{k} \end{array}$$

where the pixel average and standard deviation of the guidance image in the window $\omega_{k}$ with radius *r* and central pixel *k* are $\mu_{k}$ and $\sigma_{k}$, ${\bar{p}}_{k}$ is the mean of the filtering image in the window $\omega_{k}$, and $N_{\omega_{k}}$ is the total number of pixels in the window $\omega_{k}$.

Bring Equations (20) and (21) into Equations (18) and (19).

(22) $$\begin{array}{c} a_{k}=\frac{\sigma_{k}^{2}}{\sigma_{k}^{2}+\lambda \Gamma } \end{array}$$

(23) $$\begin{array}{c} b_{k}=\left(1-a_{k}\right)\mu_{k} \end{array}$$

After calculating $E\left(a_{k},b_{k}\right)$ in all windows $\omega_{k}$ of the image, the final output of the filter is calculated as follows:

(24) $$\begin{array}{cc} q_{i} & =\frac{1}{N_{\omega_{k}}}\sum_{i\in \omega_{k}}\left(a_{k}I_{i}+b_{k}\right)\\ \; & ={\bar{a}}_{i}I_{i}+{\bar{b}}_{i} \end{array}$$

The calculation formula of the texture layer defined by the image decomposition model is as follows:

(25) $$\begin{array}{c} l=I-q \end{array}$$

where *q* is a structure layer processed by Equation (24). The texture layer is multiplied with β.

(26) $$\begin{array}{c} l^{\prime }=\beta \cdot l=\beta \cdot \left(I-q\right) \end{array}$$

$l^{\prime }$ is the enhanced texture layer. Output image *f* is the sum of *q* and $l^{\prime }$.

(27) $$\begin{array}{c} f=q+l^{\prime } \end{array}$$

Substitute Equation (24) into Equation (26).

(28) $$\begin{array}{c} l^{\prime }=\beta \cdot \left(I-\bar{a}I-\bar{b}\right)=\beta \cdot \left(1-\bar{a}\right)\cdot I-\beta \cdot \bar{b} \end{array}$$

The gradient of the enhanced texture layer is calculated as follows:

(29) $$\begin{array}{c} ▽l^{\prime }=\beta \cdot \left(1-\bar{a}\right)\cdot ▽I \end{array}$$

Calculate the gradient of the structure layer on the basis of Equation (15), as follows,

(30) $$\begin{array}{c} ▽q=\bar{a}\cdot ▽I \end{array}$$

The gradient of the texture layer should not be larger than the gradient of the structure layer. Otherwise, the noise in the texture layer will be amplified. Therefore, the following is not equal to the establishment:

(31) $$\begin{array}{c} ▽l^{\prime }\leq ▽q \end{array}$$

Bring Equations (29) and (30) to Equation (31).

(32) $$\begin{array}{c} \beta \cdot \left(1-\bar{a}\right)\cdot ▽I\leq \bar{a}\cdot ▽I \end{array}$$

Therefore,

(33) $$\begin{array}{c} \beta \leq \frac{\bar{a}}{1-\bar{a}} \end{array}$$

When the value of β is very small, the details will be suppressed. On the other hand, noise will be amplified for larger β values. Therefore, considering the balance between noise suppression and enhancement of details, the value of β is set as follows:

(34) $$\begin{array}{c} \beta =\frac{\bar{a}}{1-\bar{a}} \end{array}$$

According to Equation (34), when $\bar{a}$ is close to 1, the value of β will be very large, and it is easy to excessively enhance the edge of the image. Therefore, the introduction coefficient γ in Equation (34) is as follows:

(35) $$\begin{array}{c} \beta ={\left(\frac{\bar{a}}{1-\bar{a}}\right)}^{\gamma } \end{array}$$

where 0≤γ≤1. In order to prevent the image from excessive enhancement, this article converts the input image to the HSV channel and only enhances the edges for the V channel, while the H component and S component remain unchanged. The formula used is as follows:

(36) $$\begin{array}{c} output_{v}=R_{v}\left(x,y\right)+\beta \cdot \left(R_{v}\left(x,y\right)-q_{v}\left(x,y\right)\right) \end{array}$$

where $R_{v}\left(x,y\right)$ is the V component of R(x,y). $q_{v}\left(x,y\right)$ is obtained by EGIF processing $R_{v}\left(x,y\right)$. $output_{v}$ is the V component of the output image. Finally, the color space of the image is transferred to RGB to obtain the night image enhanced. The edges of the image obtained by the proposed method are clear. By controlling the parameters of image enhancement, excessive enhancement of the image is avoided.

## 4. Experiment

In order to verify the superiority of the proposed algorithm in processing nighttime images, Ammen [27], Dong [28], SRIE [29], NPE [22], MF [15], Jiep [30], Ying [31], LIME [32], AIEM [7], and RBMP [33] have been chosen as the comparison algorithms. Nighttime images in the dataset of literature [34], which have a lot of dark light regions and contain artificial light sources, are selected to evaluate the proposed algorithm. Experimental results are shown for eight groups of images with different degrees of richness of texture structure in dark light regions to verify the effectiveness of the proposed algorithm in detail supplementation, edge protection, and noise suppression. All experiments in this research were run on MATLAB R2018a on a PC with 1.6 GHz CPU and 8 GB RAM. The proposed image decomposition-based enhancement algorithm is implemented as follows Algorithm 1:
**Algorithm 1** Image decomposition-based enhancement algorithm.
Step 1: Enter a low-illumination image *I*.
Step 2: Obtain the structure layer $I_{S}$ using Formula (6).
Step 3: Obtain the texture layer $I_{T}$ using Formula (7).
Step 4: Enhance the brightness of the structural layer using Formula (11).
Step 5: Stretch the structural layer saturation using Formula (12).
Step 6: Denoise the texture layer using Formula (13).
Step 7: Obtain the fused image R(x,y) using Formula (14).
Step 8: Enhance edge to fused image using Formula (36).
Step 9: Output enhanced image *output*.


In this section, we adopt an evaluation method combined with subjective evaluation and objective evaluation and use the performance of image decomposition night image enhancement methods proposed by night image verification, including noise suppression and edge details. Subjective evaluation can evaluate the quality of the results from the direction of human perception, and objective evaluation can calculate the image quality score from the statistical characteristics of various natural images.

### 4.1. Experimental Parameter Settings

**Figure 6:**
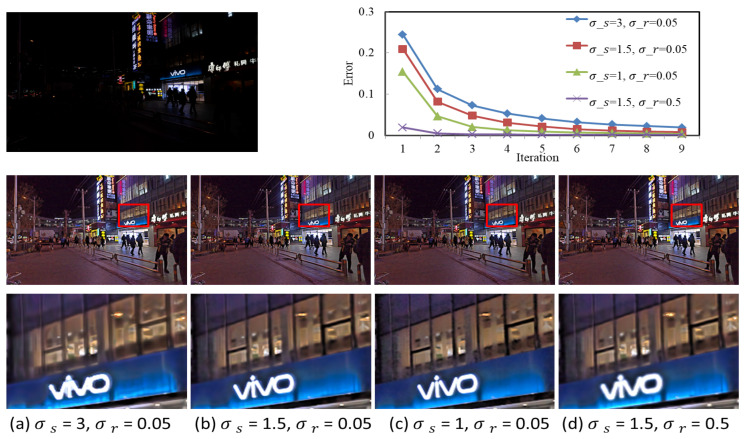
Enhancement effect for different $\sigma_{s}$ and $\sigma_{r}$. First row: convergence curves of ${∥I_{s}^{t+1}-I_{s}^{t}∥}_{F}/{∥I_{s}^{t}∥}_{F}$ for the original image and different parameters. Second row: the resultant image after enhancement. Third row: local enlargement image.

The results of the comparison algorithms are generated by the code downloaded from the author’s website, and the parameters are set as in the paper. The proposed algorithm uses a rolling guide filter to decompose the image. The decomposition effect is related to $\sigma_{s}$, $\sigma_{r}$, and the number of iterations *t*. The effect of different parameter settings is shown in Figure 6. ${\left\|\cdot \right\|}_{F}$ represents Frobenius norm, and the closer the value of the Frobenius norm of the two images, the more similar the two pictures are. The convergence curve shows that generally an optimal solution can be obtained within four iterations even with different parameter settings. Thus, for all experiments, we let t=4. It is seen from Figure 6 that the larger the $\sigma_{s}$ and $\sigma_{r}$, the smoother the images. When the $\sigma_{s}$ and $\sigma_{r}$ are too large, the image is blurred and the edges are lost, as shown in the lights of Figure 6a,d. If *s* is too small, it will cause noise residue, as shown in the window in Figure 6c. Therefore, this article sets $\sigma_{s}=1.5$, $\sigma_{r}=0.05$.

**Figure 7:**
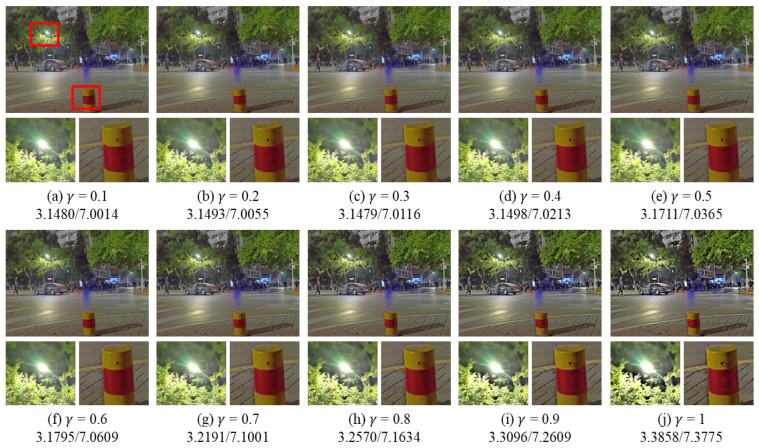
Enhancement results for different γ. A/B: A represents the CEIQ value, B represents the image discrete information entropy.

The proposed algorithm uses the effective guidance filter (EGIF) to enhance the edges, and the enhancement strength is related to γ. The value range of γ is usually [0, 1], and different γ will obtain different edge enhancement results. As shown in Figure 7, in general, the larger γ is, the greater the contrast and information entropy of the image, indicating sharper edges and richer details. However, too much γ will cause the over-enhancement phenomenon of the image, resulting in an unnatural image. Taking this into account, the value of γ is set to 0.9 in this paper.

### 4.2. Subjective Comparison Experiments of Noise Suppression and Edge Detail Retention

The subjective evaluation method is still the main evaluation method to evaluate the performance of image enhancement methods, especially for the lack of reference images such as night image enhancement methods. The subjective perception of vision is more reflective of the overall image quality improvement, including image contrast, noise suppression, and detail retention, as well as the naturalness of the enhanced resultant image.

**Figure 8:**
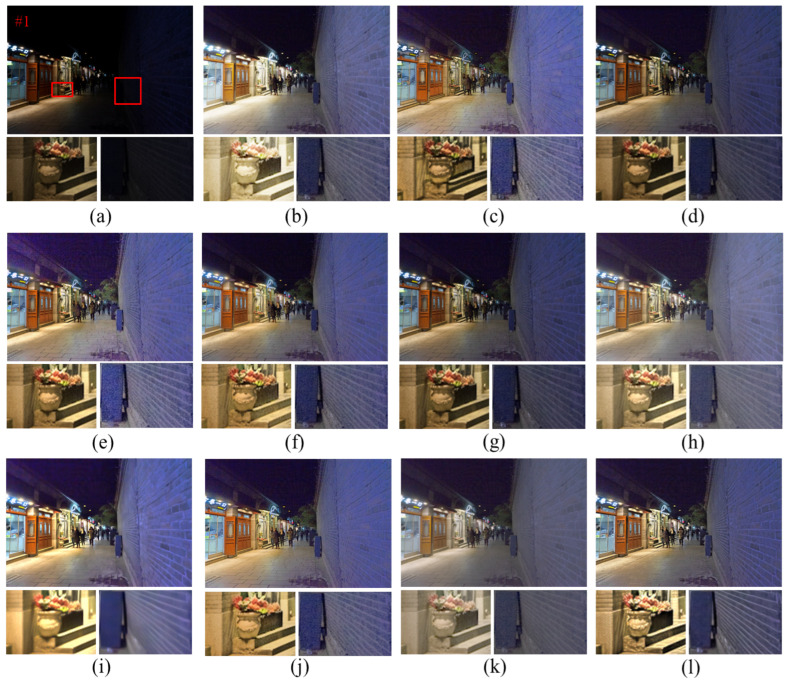
Comparison of different enhancement methods for nighttime images. (**a**) Original image. (**b**) Ammen. (**c**) Dong. (**d**) SRIE. (**e**) NPE. (**f**) MF. (**g**) Jiep. (**h**) Ying. (**i**) LIME. (**j**) AIEM. (**k**) RBMP. (**l**) Ours.

Figure 8 shows the enhancement results of the nighttime images by different algorithms, and the details are marked with red boxes and enlarged on the enhanced images. From the analysis of Figure 8, it can be seen that the SRIE and Jiep algorithms fail to improve the overall brightness of the image in terms of dark light enhancement. In terms of noise suppression, combined with the enlarged “wall” in Figure 8, it can be observed that the enhancement results of Ammen, Dong, NPE, MF, and AIEM algorithms all contain a large amount of noise, which seriously affects the image quality. The Jiep algorithm adds denoising processing and achieves noise suppression by smoothing the reflective layer of the image, but the brightness of the enhanced image obtained by the Jiep algorithm is darker. In terms of color correction, the RBMP algorithm has more severe color distortion, such as the overall color distortion of the enlarged vase and wall in Figure 8k. In terms of detail protection, LIME algorithm loses the detail information of the image due to the over-smoothing of the image caused by denoising, such as the blurred texture of the wall shown in Figure 8i. The proposed algorithm improves the brightness of the image while ensuring the effect of image noise suppression, the noise of the wall is smoothed, and the edge texture information of the wall is preserved with clear image edge contours.

**Figure 9:**
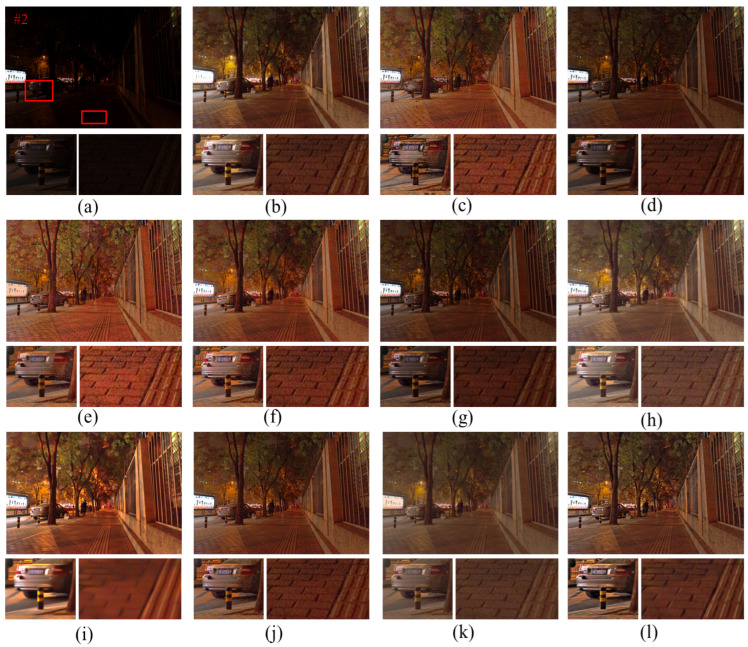
Comparison of different enhancement methods for nighttime images. (**a**) Original image. (**b**) Ammen. (**c**) Dong. (**d**) SRIE. (**e**) NPE. (**f**) MF. (**g**) Jiep. (**h**) Ying. (**i**) LIME. (**j**) AIEM. (**k**) RBMP. (**l**) Ours.

As observed in Figure 9, the Dong algorithm boosts the brightness of the image in terms of luminance enhancement but produces an over-enhancement phenomenon in the high-brightness areas of the image, such as the light sign shown in Figure 9c, where some details are lost due to excessive brightness. The SRIE and Jiep algorithms do not improve the brightness of the image enough, and the average brightness of the image is low. The proposed algorithm makes the image brighter while avoiding overexposure of the bright areas, which significantly improves the visibility of the image. In terms of noise suppression, the LIME algorithm has a good denoising effect, but the image details are blurred due to over-smoothing. For example, the details of the car in Figure 9i are missing more seriously, the ground texture is lost, and the edges are blurred. The proposed algorithm enhances the edge details of the image compared with the LIME algorithm, and the edges are more prominent, which improves the clarity of the image and has a better image denoising effect.

**Figure 10:**
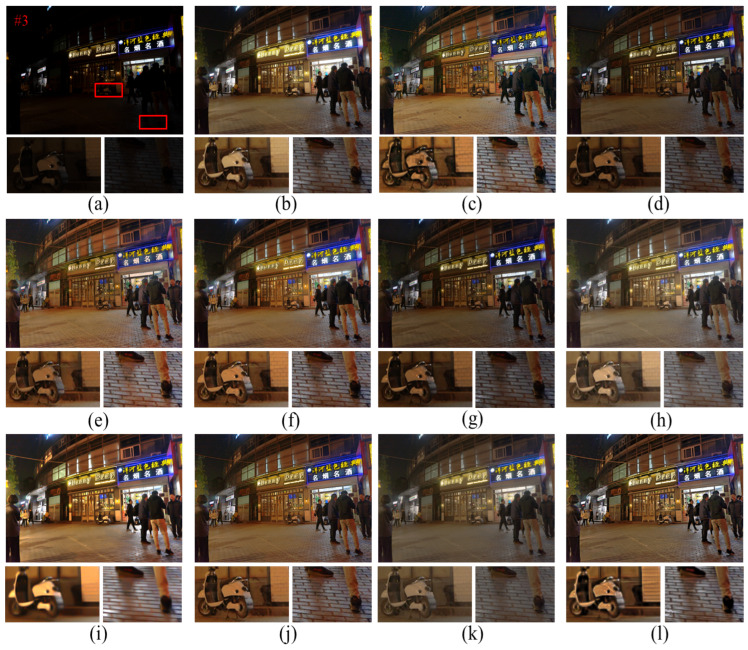
Comparison of different enhancement methods for nighttime images. (**a**) Original image. (**b**) Ammen. (**c**) Dong. (**d**) SRIE. (**e**) NPE. (**f**) MF. (**g**) Jiep. (**h**) Ying. (**i**) LIME. (**j**) AIEM. (**k**) RBMP. (**l**) Ours.

As shown in Figure 10, the Ammen, NPE, MF, and AIEM algorithms improve the image brightness but, at the same time, significant noise appears, such as the presence of obvious noise points around the vehicle in Figure 10, indicating that these algorithms lack effective noise suppression. The RBMP algorithm processes images with high brightness, resulting in loss of details and the overall white color of the image due to the lack of color correction of the image. The Ying algorithm does not correct the color of the image and also shows some color distortion. The texture details of the image processed by the LIME algorithm are obviously lost. As shown in the enlarged floor image in Figure 10i, the texture of the floor tiles is blurred, leading to difficulties in extracting line features. In terms of texture details, the proposed algorithm is able to improve the image brightness and contrast while being able to maintain the image detail information due to the inclusion of edge enhancement. As shown in Figure 10l, the edges of the electric car are more clearly defined, which proves that the proposed algorithm makes the image richer in detail information and the visual effect of the image is greatly improved.

**Figure 11:**
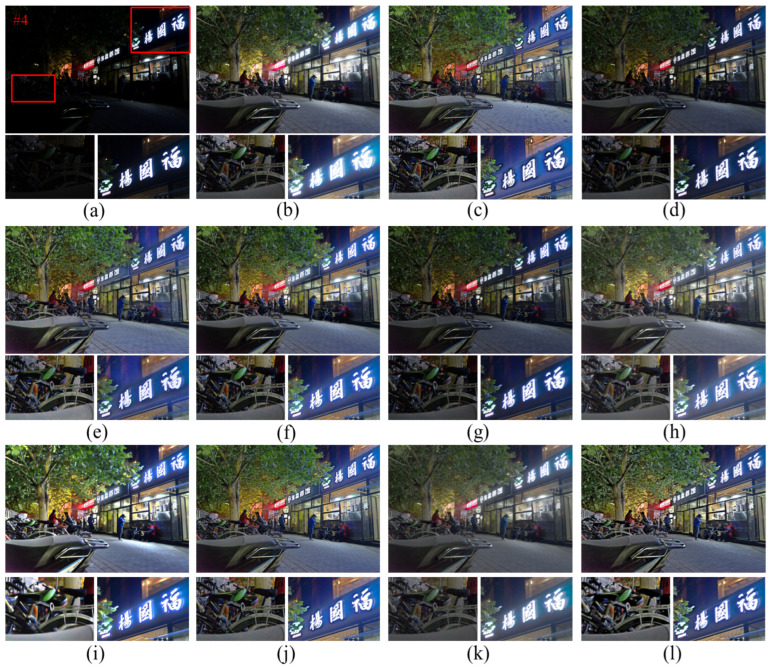
Comparison of different enhancement methods for nighttime image. (**a**) Original image. (**b**) Ammen. (**c**) Dong. (**d**) SRIE. (**e**) NPE. (**f**) MF. (**g**) Jiep. (**h**) Ying. (**i**) LIME. (**j**) AIEM. (**k**) RBMP. (**l**) Ours.

From the observation of Figure 11, it can be seen that the RBMP algorithm shows a fading phenomenon, and many objects have white color and more serious fading phenomenon. The proposed method can better maintain the light distribution and color information of the image. As shown in the enlarged image of the light sign in Figure 11l, the image brightness is moderate and there is no over-enhancement. The saturation of the image is in accordance with the visual effect of human eyes, and the image is more natural. As can be observed from the partial zoomed image of the bicycle, the image processed by the proposed algorithm retains more edge texture information and the texture of the seat is clearly visible, so the proposed algorithm has a better edge preservation effect than other comparative algorithms.

**Figure 12:**
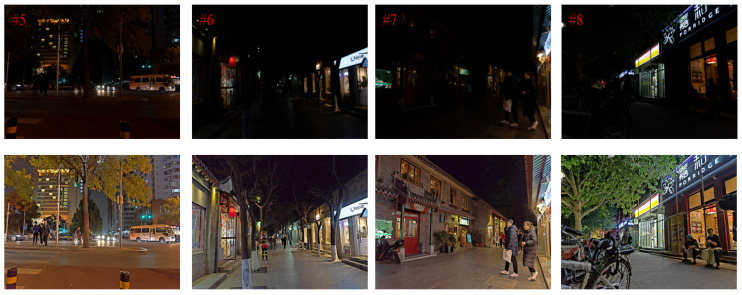
More night image enhancement results.

In Figure 12, more nighttime image enhancement results are shown in this paper to demonstrate the effectiveness of the proposed nighttime image enhancement method. Comparing the input image and the enhanced image, it can be seen that the brightness of the enhanced image is improved, the detail information in the dark areas that is difficult to distinguish but contains rich structure becomes clear, the overall visual effect of the image is improved, and the image noise is suppressed while the texture edge information is protected.

### 4.3. Objective Comparison Experiments of Noise Suppression and Edge Detail Preservation

**Figure 13:**
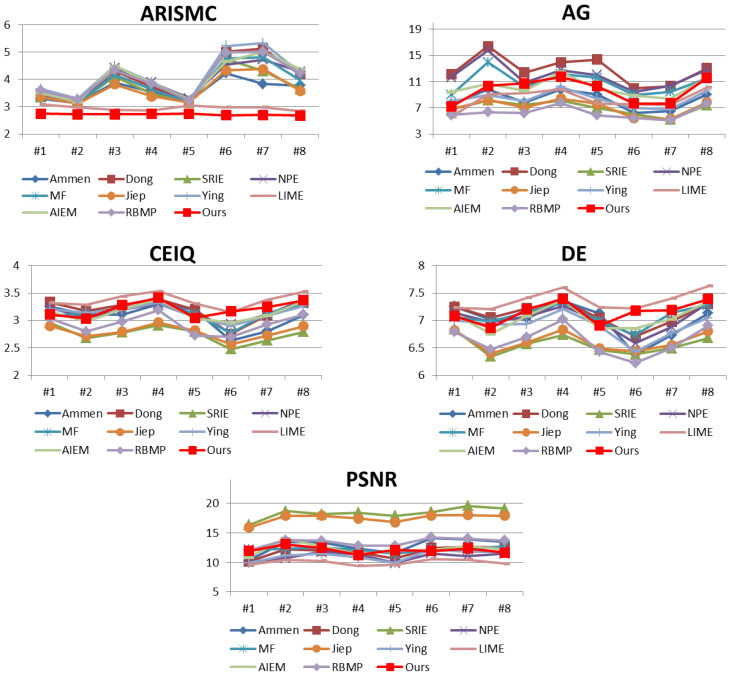
Comparison of objective evaluation metrics for 8 enhanced nighttime images.

In order to objectively reflect the enhancement effect of each algorithm in processing low-light images, ARISMC [35], AG (average gradient), CEIQ [36], DE (discrete information entropy) [37], and PSNR (peak signal-to-noise ratio) are used in this paper to measure the quality of the enhanced nighttime images. DE is used to represent the information content of an 8-bit image. The resolution of the histogram is usually 256. Discrete entropy is a statistical measure of stochasticity, the maximum value of the information entropy of an image is eight, and the closer it is to eight implies that the image contains more details. The formula is shown as follows.

(37) $$\begin{array}{c} \mathrm{DE}=-\sum_{i=0}^{255}p\left(i\right)\times \log_{2}\left(p\left(i\right)\right) \end{array}$$

where p(i) represents the probability of gray value *i*. Figure 13 lists the comparison of objective evaluation metrics of different algorithms for processing nighttime images.

From Figure 13, it can be seen that the proposed algorithm outperforms the other algorithms in ARISMC. The average gradient value of the proposed algorithm only lags behind Dong, NPE, and MF algorithms, indicating that the proposed algorithm can effectively improve the sharpness of images. The CEIQ index only lags behind LIME, indicating that the proposed algorithm achieves good results in the improvement of nighttime image contrast, which reflects the effectiveness of the proposed algorithm. Discrete entropy is a statistical measure of randomness, and higher entropy value usually indicates more details. The proposed algorithm only lags behind the LIME algorithm in DE index, which indicates that the nighttime images processed by the proposed algorithm are enriched in detail content.

The proposed algorithm does not perform well on the PSNR index, which is a reference measure of image noise and distortion. However, due to the lack of good quality reference images for nighttime images, the original nighttime image is used as the reference image in this paper. However, due to the low brightness and high implied noise of the original night image, the use of the PSNR metric cannot evaluate the denoising effect of the algorithm well. Therefore, we use the SNR (signal-to-noise ratio) metric to continue to verify the image denoising effect. In a large homogeneous area, the ratio of the mean value of image elements to the standard deviation can be considered as the signal-to-noise ratio of the image. The more homogeneous the image is, the closer the estimated value is to the true value [38]. The SNR expression is as follows.

(38) $$\begin{array}{c} \mathrm{SNR}=\frac{\mathrm{mean}(I)}{\sqrt{\frac{1}{m*n}*\sum_{i=1}^{m}\sum_{j=1}^{n}{\left(I\left(i,j\right)-\mathrm{mean}\left(I\right)\right)}^{2}}} \end{array}$$

where m∗n represents the size of the image, I(i,j) represents the pixel value of the *i*-th row and *j*-th column, and mean(I) represents the mean value of the image.

**Figure 14:**
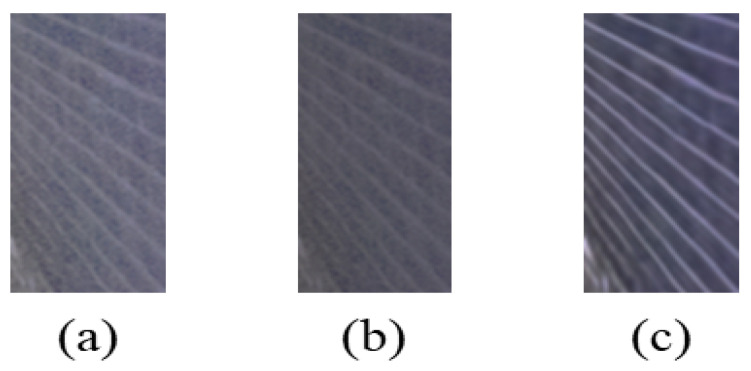
The results of the three algorithms after processing. (**a**) Ying. (**b**) RBMP. (**c**) Ours.

We intercept part of the uniform region in Figure 8 for testing, and the test results are shown in Table 1. The proposed algorithm has a low SNR value, and the Ying and RBMP algorithms have a high SNR value. We show the effect graph of these three algorithms as shown in Figure 14, and we can see that the denoising effect of the Ying and RBMP algorithms is not good. The noise of the processed image by the proposed algorithm disappears, but the SNR index is the lowest instead. We explain it as follows. In the proposed algorithm, the method used to enhance the image contrast consists of two parts: gamma correction before denoising the texture layer and edge enhancement at the end. To verify the effect of contrast on the SNR index, we process the images with and without gamma correction and edge enhancement, respectively, and then calculate the SNR values. The results are shown in Table 2. The SNR value after removing these two steps reaches 16.5549, which is the largest value in the comparison algorithm. Gamma correction and edge enhancement work on contrast enhancement but will amplify noise if noise removal is not effective. However, in terms of the effect of the image shown in Figure 14c, the proposed algorithm has a good effect on noise removal and does not amplify the noise, and the image has a high contrast and clear edge texture. In contrast, the images processed by the Ying and RBMP algorithms have noise visible to the naked eye and low contrast. Therefore, we suppose that the reason for the low SNR value of the images processed by the proposed algorithm is the increase in contrast, not the high noise. It further verifies the effectiveness of the algorithm in this paper in improving the contrast and suppressing the noise.

Overall, the results of the comparison experiments show that the proposed algorithm improves the image contrast and brightness while enhancing the detailed texture and suppressing the noise in dark areas. The proposed algorithm processes images with clear details, effectively improves the quality of low-illumination images, and better preserves the edges of objects.

### 4.4. Comparison with Illumination Images

**Figure 15:**
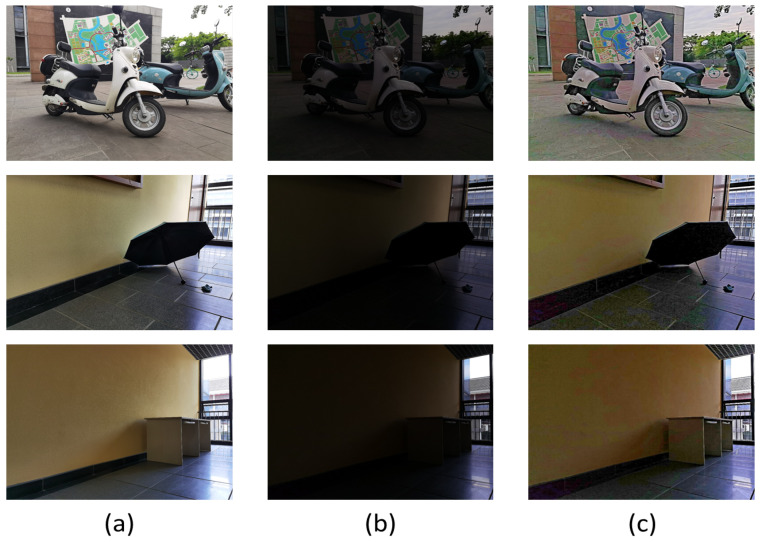
Comparison between enhanced images and illuminated images. (**a**) Illuminated image. (**b**) Low-light image. (**c**) Image enhanced by proposed algorithm for low-light image.

In order to illustrate more intuitively the effectiveness of the proposed algorithm in detail recovery and brightness enhancement of the image, the low-light image is enhanced and compared with the corresponding illuminated image. The experiment is performed using three sets of images: ’vehicle’, ’umbrella’, and ’table’. The experimental results are shown in Figure 15. The objective evaluation results are shown in Table 3. As seen in Figure 15, the proposed algorithm is able to recover the scene in the dark light image with appropriate brightness and no overexposure or under-enhancement, which is similar to the illuminated image. From Table 3, it can be seen that the image processed by the proposed algorithm has more obvious improvement in DE and CEIQ values, which is close to the illuminated image.

### 4.5. Comparison of Line Feature Extraction

**Figure 16:**
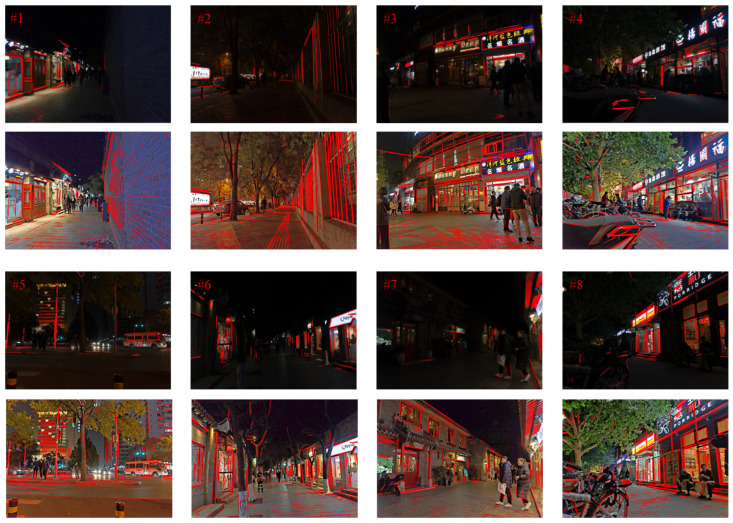
Comparison of line feature extraction for nighttime images. First row: original image; second row: proposed algorithm; third row: original image; fourth row: proposed algorithm.

**Figure 17:**
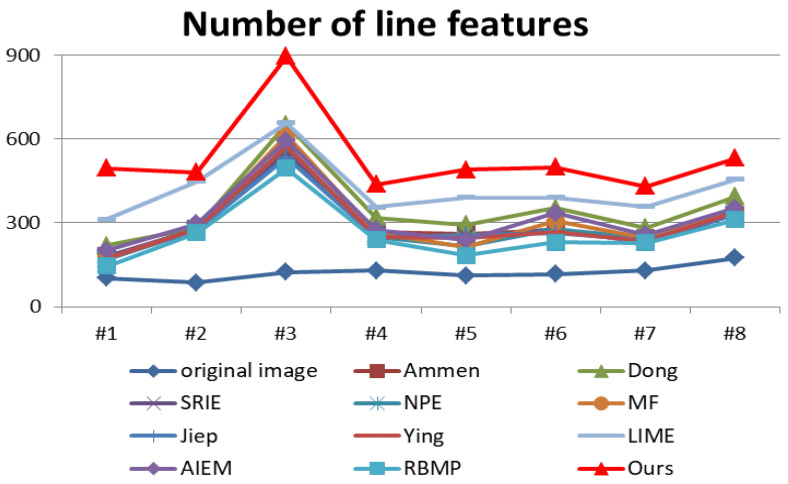
Comparison of the number of line features extracted by nighttime image enhancement algorithms.

The night image is processed by the enhancement algorithm, which supplements the detailed features of the dark light area of the image and contributes greatly to the line feature extraction of the image. The effect of line feature extraction of the night image is shown in Figure 16. The comparison data of the number of line features with other enhancement algorithms are shown in Figure 17.

As shown in Figure 16, the processing of the nighttime image by the proposed algorithm enhances the brightness of the dark light region of the image and suppresses the image noise. Due to the image texture enhancement algorithm, the texture edge details of the nighttime image are clearer, the detail information hidden in the dark light region of the image is also restored, and the number of extracted line features is substantially increased. The richness of line features in the dark light region of the image is beneficial to nighttime image stitching.

Figure 17 shows the comparative effect of the proposed algorithm and the comparison algorithm in terms of online feature extraction capability. The results show that the proposed algorithm is significantly better than other comparison algorithms in terms of enriching image structure and line feature extraction, which proves the effectiveness of the proposed algorithm in image feature extraction and helps to improve the success rate of nighttime image stitching.

### 4.6. Comparison of Nighttime Images for Stitching

**Figure 18:**
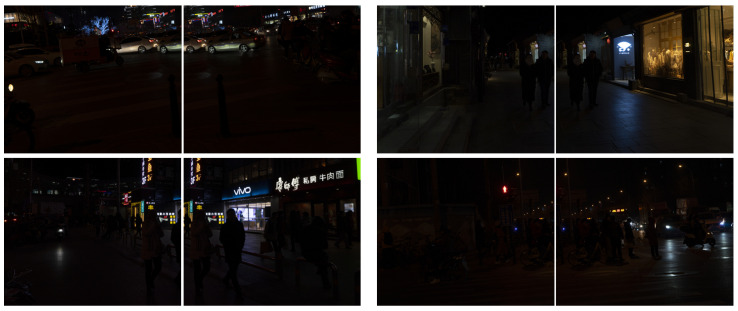
Experimental images of night stitching.

**Figure 19:**
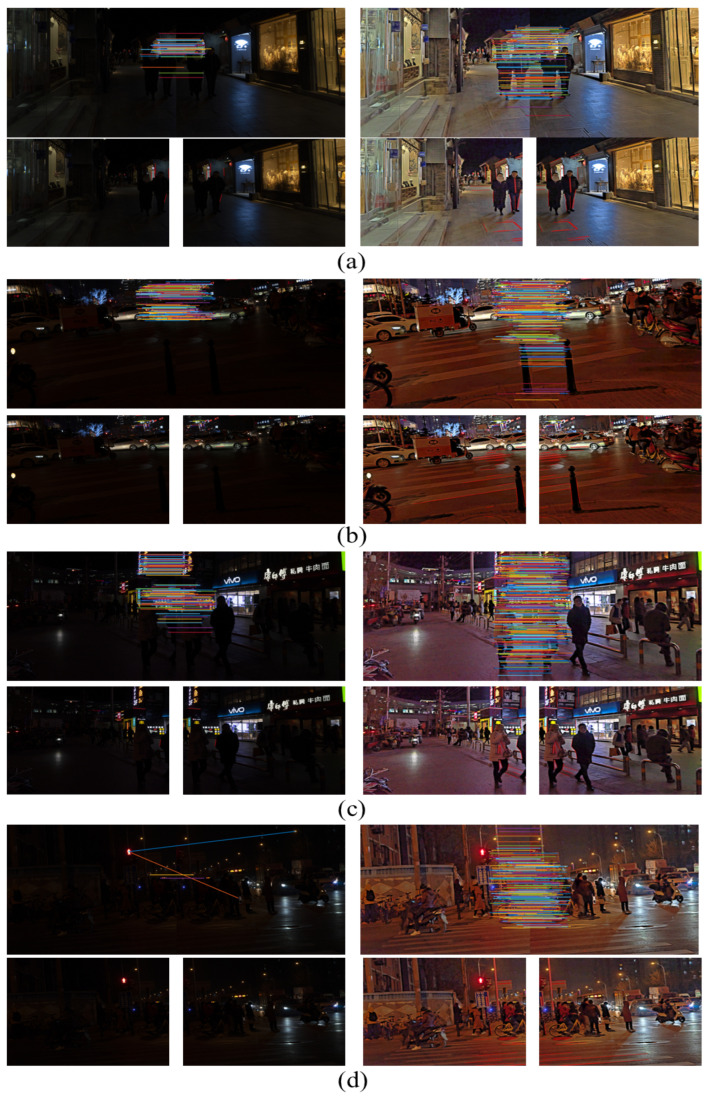
Comparison of the matching effect of point and line features before and after image enhancement in four groups. The first column: comparison of point feature matching; the second column: comparison of line feature matching.

In this paper, the nighttime images from the dataset [34] are clipped and segmented into two images with overlapping regions for stitching experiments. We extract feature points by using the SIFT algorithm, eliminate the mis-matched pairs applying the RANSAC algorithm, extract line features using the LSD algorithm, and generate the stitched images by the linear fusion method. The four sets of images to be stitched are shown in Figure 18. The comparison effect of matching point and line features for the four groups of images to be stitched before and after enhancement is shown in Figure 19.

As can be seen from Figure 19, the number of extracted and aligned line features and point features of the image is increased after processing by the proposed enhancement algorithm. As shown on the pavement of Figure 19b, the details of the dark light areas of the image processed by the proposed enhancement algorithm are visible, and the number of line features and point features extracted and aligned is increased. As shown in Figure 19d, mis-matched pairs are caused due to the presence of faint bright spots in the nighttime images, which directly led to stitching failure. As seen in Figure 19d, the pre-processing of the nighttime image by the proposed enhancement algorithm increases the number of feature point pairs in the image and no mismatched pairs appear.

**Figure 20:**
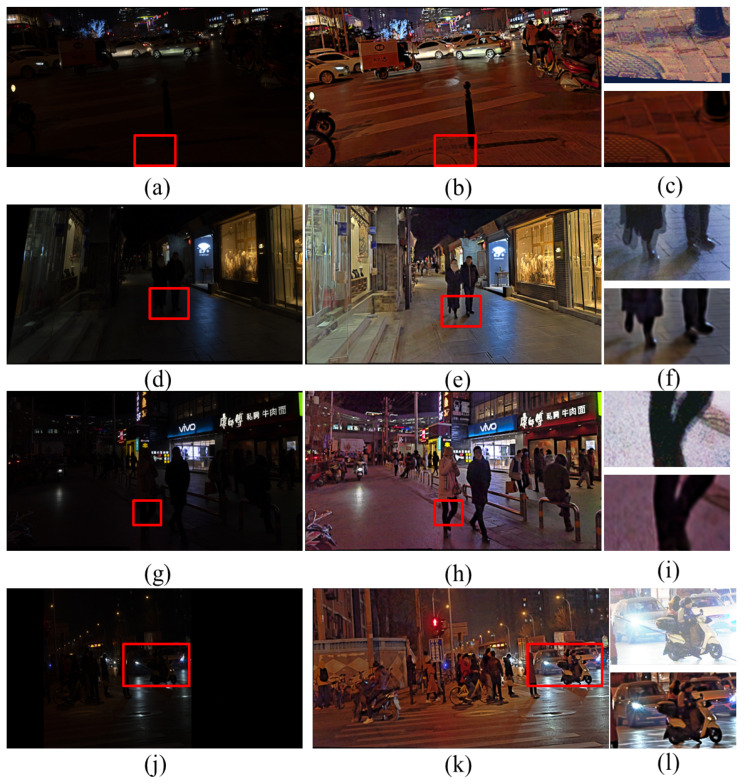
First column: stitching of nighttime images; second column: stitching of nighttime images after enhancement; third column: local enlargement.

In order to make the details of the night stitched image visible and easy to compare, we enhance the brightness of the stitched night image by the histogram equalization method, and the effect is shown in the enlarged figure in Figure 20. The effect of night image stitching and the effect of night image stitching after the enhancement process are shown in Figure 20. The objective quality of the stitched images is evaluated using AG (average gradient), DE (discrete information entropy), and CEIQ. The results are shown in Table 4, and the image names in the table are consistent with the image names in Figure 20.

From Figure 20c,f,i, it can be seen that when stitching the nighttime images directly, the stitched images obtained are misaligned and overlapped due to the lack of feature information. Figure 20j shows that this group of nighttime images failed to be stitched together, which is highly likely to cause image stitching failure due to the small number of feature points and the presence of mis-matched pairs. Table 4 shows that the stitched images (b,e,h,k) after the enhancement process are significantly improved in terms of image sharpness and contrast. These four sets of stitched images show that the line features and point features are complemented by the enhanced nighttime stitched images, which results in a more accurate transformation matrix, and the ghosting misalignment disappears. Meanwhile, The quality of the stitched images is improved. This experiment proves that the proposed enhancement algorithm is suitable for nighttime image stitching.

## 5. Conclusions

Aiming at the problem of insufficient line feature extraction during the splicing process of night images, a nighttime image stitching method based on image decomposition enhancement is proposed. The proposed method decomposes the image using the rolling guidance filter to obtain a higher quality structural layer. The structural layer brightness is enhanced using an improved enhancement function, and the texture layer is denoised using the BM3D algorithm. After the enhanced structure layer and the smoothed texture layer are fused, the fused night image is then contrast enhanced by the edge enhancement function, and finally a higher quality and more detailed night enhanced image is obtained.

In this paper, the proposed algorithm is validated using nighttime images and compared with ten other enhancement algorithms. From the experimental results, we can see that the nighttime images processed by the proposed enhancement algorithm are processed to obtain images with rich details, clear textures, and natural colors. By comparing the enhanced images with other algorithms for line feature extraction, the proposed algorithm has a more obvious advantage in the number of line features extracted. In addition, through the image stitching experiments, it is proved that the proposed algorithm improves the efficiency of nighttime image stitching and reduces the phenomenon of misalignment and ghosting. In summary, the proposed nighttime image stitching method based on image decomposition enhancement can improve the quality of nighttime images, supplement texture information, and increase the number of line features in dark light areas of images, resulting in efficient and accurate stitching, which provides value for nighttime security surveillance applications. 

## Figures and Tables

**Table 1:** SNR evaluation results of locally enhanced images of #1.

Methods	Ammen	Dong	SRIE	NPE	MF	Jiep	Ying	LIME	AIEM	RBMP	Ours
SNR	9.1678	7.7301	7.7478	7.5599	8.5608	7.6108	13.4639	11.8873	9.6383	15.8182	5.0489

**Table 2:** SNR evaluation results of the local image enhanced by the proposed algorithm for image #1.

Methods	Ours (without Gamma Correction)	Ours (without Gamma Correction and Edge Enhancement)
SNR	11.3355	16.5549

**Table 3:** The assessment results on the ’vehicle’, ’umbrella’ and ’table’ images.

Image Name	Image Index	AG	DE	CEIQ
vehicle	(a)	9.5308	7.5610	3.5549
	(b)	2.3725	5.7367	2.1842
	(c)	8.9704	7.4100	3.3822
umbrella	(a)	10.7415	7.6450	3.5293
	(b)	1.7601	5.5031	2.1237
	(c)	4.0315	6.9612	3.0986
table	(a)	7.2880	7.2186	3.2175
	(b)	2.0186	5.3812	2.2509
	(c)	3.8058	6.6435	2.9434

**Table 4:** Objective evaluation of stitched images.

	Images	(a)	(b)	(d)	(e)	(g)	(h)	(j)	(k)
Metric									
AG		2.5148	8.9469	2.3347	7.9535	2.9061	8.4843	1.1548	7.1729
DE		5.6975	7.3219	4.2336	6.4785	4.5531	7.0246	3.3175	6.8408
CEIQ		2.1882	3.3638	1.8909	2.6831	1.9174	3.1295	1.8664	3.0218

## Data Availability

The data presented in this study are available on request from the corresponding author. The data are not publicly available due to privacy.
